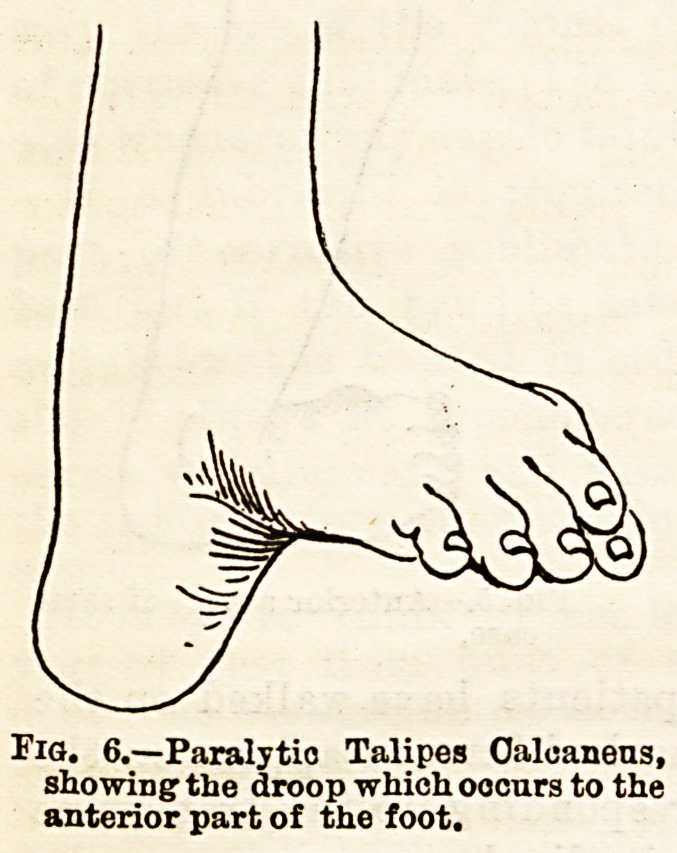# Club Foot.—II

**Published:** 1895-04-13

**Authors:** Walter L. Woolcombe

**Affiliations:** Assistant Surgeon South Devon and East Cornwall Hospital


### CLUB-FOOT.—II.

By Walter L. Woolcombe, F.R.C.S.E., Assistant Surgeon South Devon and East Cornwall Hospital.

(*Continued from page* 403, *Vol. XVII.*)

The second part of the subject deals with the different forms assumed, and a very few words will suffice for this, as everyone is familiar with the names given to them, and there is no time in a short paper like this to go minutely into the anatomical arrangement of the parts in each different variety, for if we once master the order in and the extent to which the different structures are involved in the congenital and acquired forms of one variety we have a key to all the others.

There are four primitive forms of club-foot. Talipes equinus, T. calcaneus, T. varus, and T. valgus, and four compound forms are usually described Equino-varus and Equino-valgus: Calcaneo-varus and Calcaneo-valgus; and each of these may exist as a congenital or acquired deformity, though with varying frequency, as will be shown when I give a short description of each, though I do not propose to lay very much stress on the difference between the two varieties for this reason—that, according to the theory which I have adopted to explain the large majority of cases, both congenital and acquired, namely, disordered nervous action, the actual starting-point is similar in both varieties, and in either case the spasm may end in—(*a*) Complete and speedy resolution; (*b*) long-continued spasm, never completely resolving; or (*c*) paralysis; and the only difference lies in the fact that in the congenital variety the lesion has taken place when the different parts of the body are growing and developing at a very much greater pace in proportion to the total length of the fœtus than they do at any subsequent stage of the child's growth, and consequently a spasmodic affection of a muscle in the fœtus lasting, we will say, a week, and preventing during that time the growth of that muscle, will leave a permanent mark on the structures to which it is attached, which could not be produced by a much more prolonged affection of the muscle had it taken place when the child was, we will say, a few years old, so that it may be laid down as an axiom that the severity and amount of deformity will vary in inverse proportion to the age of the child when the lesion took place, and the difficulty of correcting it will increase in the same proportion. And now to briefly consider the different forms:—

**Talipes Equinus** is very seldom found as a congenital form, and it was even held for a long time to be always acquired, but several well-authenticated cases have now been observed, of which Mr. Adams has recorded two or three It is, however, very common as an acquired deformity, and of 3,000 cases of club-foot treated at the Royal Orthopædic hospital 170 were of this variety, not including the compound forms. It may result from (*a*) spasmodic contraction of all the muscles of the leg, the extensors of the foot being the more powerful and gradually overcoming the flexors; (*b*) paralysis of the flexors, the unopposed extensors gradually becoming shortened in adaptation to the altered state of things; (*c*) long continued retention of the foot in one position from pressure of the bedclothes during a long illness; (*d*) contraction of cicatrices in the calf from burns, abscesses, lacerated wounds, &c.

Fig. 1. — Spasmodic Talipes Equinus. Toes claw-like from action of ext. longus digitorum. (After Adams.)

In a fully developed case the heel is drawn up and the toes pointed, so that the phalanges alone rest on the ground in walking or, rather, the weight is borne on the heads of the metatarsals; but here there is a difference according as to whether the case is spastic or paralytic. If the muscles on the front of the leg retain their power the phalanges will rest flat on the ground or be drawn up into a claw-like con-

Figs. 2 and 3.—Case of Paralytic Talipes Equinus.
In Fig. 2 the weight is borne on the heads of metatarsals. Fig. 3 is a later stage where from much use the weight has come to be borne on the astragalus. (After Noble Smith.)

dition according to the amount of contraction in the ext. longus digitorum; if paralytic, the toes will droop, catch in everything in walking, and be doubled back under the foot, so that the weight comes gradually to be borne on the dorsum of the feet, and finally on the astragalus, which is always prominent. Of course, the spasmodic case may occupy any position between complete extension and slight contraction only of the calf muscles, but frequently there is a tendency for the contraction to stop at a point which allows the foot to be flexed to a right angle with the leg, but no farther, and this degree has come to be known as right-angled contraction of the tendo Achillis.

In spastic cases, where walking is continued, the anterior part of the foot becomes widened, the plantar fascia contracted, the astragalus prominent, and the leg small from retarded development.

The downward displacement of the anterior part of the foot depends originally upon the contraction of the calf muscles acting on the os calcis, and on this alone; but subsequently the arch becomes contracted, and the plantar fascia and deep ligaments so shortened that the deformity eventually depends as much upon a bend of the foot at the transverse tarsal joint, as on the contraction of the tendo Achillis.

Where the contraction of the transverse tarsal joint is great the scaphoid may be in contact with the tibia, and the fifth metatarsal has also been described as almost touching the same bone.

The diagnosis is evident, except in slight cases, where the only symptom is slight lameness; and in ex.mining these cases it is important to raise the leg, at the same time keeping the knee straight; if the knee is allowed to bend it relaxes the gastrocnemius, and a slight degree of equinus disappears.

**Talipes Varus.**—This is the most frequent of the congenital forms, and consequently the worst cases we have to treat are of this variety, and I shall therefore make most of my remarks on congenital forms apply to it, and say little about the remaining ones. In a moderately severe case the part of the foot anterior to the transverse tarsal joint is turned in towards the opposite foot. The inner edge of the foot is drawn up. The os calcis is drawn upwards posteriorly and the bone has an oblique direction, the anterior part pointing more inwards and forwards than is normal. The sole of the foot is shortened by contraction of the plantar fascia, and this and the foregoing conditions give the anterior part of the sole of the foot an almost vertical position, and cause it to look backwards. The heel is small and ill-formed; the internal malleolus is less apparent than usual, and appears improperly developed, but this, and the fact that the external malleolus appears too far back, is due to the oblique position of the os calcis, which also makes the tendo Achillis appear too near the outside of the ankle. The astragalus is particularly prominent on the dorsum, and its neck (in cases that have originated in early fœtal life) is bent inwards. Owing to its forward displacement, the anterior third or more of its superior articulating surface becomes sub-cutaneous on the dorsum. The scaphoid is drawn inwards, backwards, and upwards by the tibials, so that its internal border is close to or in contact with the internal malleolus. The cuboid is as a rule little displaced. All or some of the bones may be ill-developed, but this depends, as pointed out before, on the early period of development at which they were displaced, and to their continuing to develop in this false position.

Fig. 4.—Severe case of Talipes Equino-varus. Posterior aspect.

Fig. 5.—Anterior aspect of same case.

In those cases where patients have walked on the deformed feet, two well-marked furrows appear on the sole—one transverse corresponding to the transverse tarsal joint, and one longitudinal consequent on the weight of the body rolling in the two outer metatarsals. The ligaments on the inner side are all shortened in adaptation to the position of the bones, and those on the outer side correspondingly lengthened. The muscles which are principally contracted are the tib. anticus and posticus, gastrocnemius and soleus and flexor long. digitorum.

As will have been gathered from the remarks which have been made on the causation of congenital club-foot, the muscles may be found either in a state of spasm or in a healthy condition, the spasm having subsided; or some groups may be paralysed; however, it must be admitted that as a rule they are fairly healthy, so that when the foot is replaced in a normal position they are enabled to resume their function. This is one of the principal arguments against the spasmodic theory of origin.

**Talipes Valgus** is nearly always an acquired, but is sometimes a congenital deformity. The large majority of cases of talipes valgus are due simply to a relaxed condition of all the structures which should support the arch of the foot, occurring in badly nourished patients between the ages of twelve and twenty, who have to stand long hours and carry heavy weights; and these should properly be placed in a different class ("flat-foot") as they do not really conform to the definition of club-foot. In real talipes valgus the causes are the same as in the two forms already described. The arch of the foot is flattened; the inner margin approaches or rests on the ground, while the outer is raised; and the anterior portion of the foot is everted. Sometimes the sole is turned backwards as well as out. There is not much bony deformity. The tuberosity of the os calcis is raised; the astragalus tilted forwards and downwards. The scaphoid is rotated, the internal part being depressed while the outer is raised, and the cuboid is slightly rotated outwards. By these means the arch of the foot is obliterated, and two prominences present on the inner side, *i.e.*, the tubercle of the scaphoid and the exposed head of the astragalus. The contracted muscles are the peronei and sometimes the ext. long. digitorum, whilst the ext. pollicis and abductor min. digiti may also be implicated.

**Talipes Calcaneus** is the rarest form of congenital talipes. In addition to the usual causes it may result from overstretching of the tendo Achillis after division. If resulting from spasm, the muscles implicated are the ext, proprius pollicis, ext. long. digitorum, tib anticus, and peroneus tertius. If the spasm affects all the muscles of the leg, of course talipes equinus results, the extensors of the foot being the more powerful. In the spasmodic form the whole foot is raised, the toes pointing upwards; but in the paralytic form the anterior portion of the foot generally droops from the transverse tarsal joint, and a deep groove is formed across the sole, the whole foot presenting very much the appearance produced by Chinese ladies by systematic cramping of their feet into small shoes.

Fig. 6.—Paralytic Talipes Calcaneus, showing the droop which occurs to the anterior part of the foot.

Of the compound forms I will not detain you with any description. They are merely slight varieties of the deformities already described—talipes varus and valgus being generally associated with a slight degree of equinus.

## Figures and Tables

**Fig. 1 f1:**
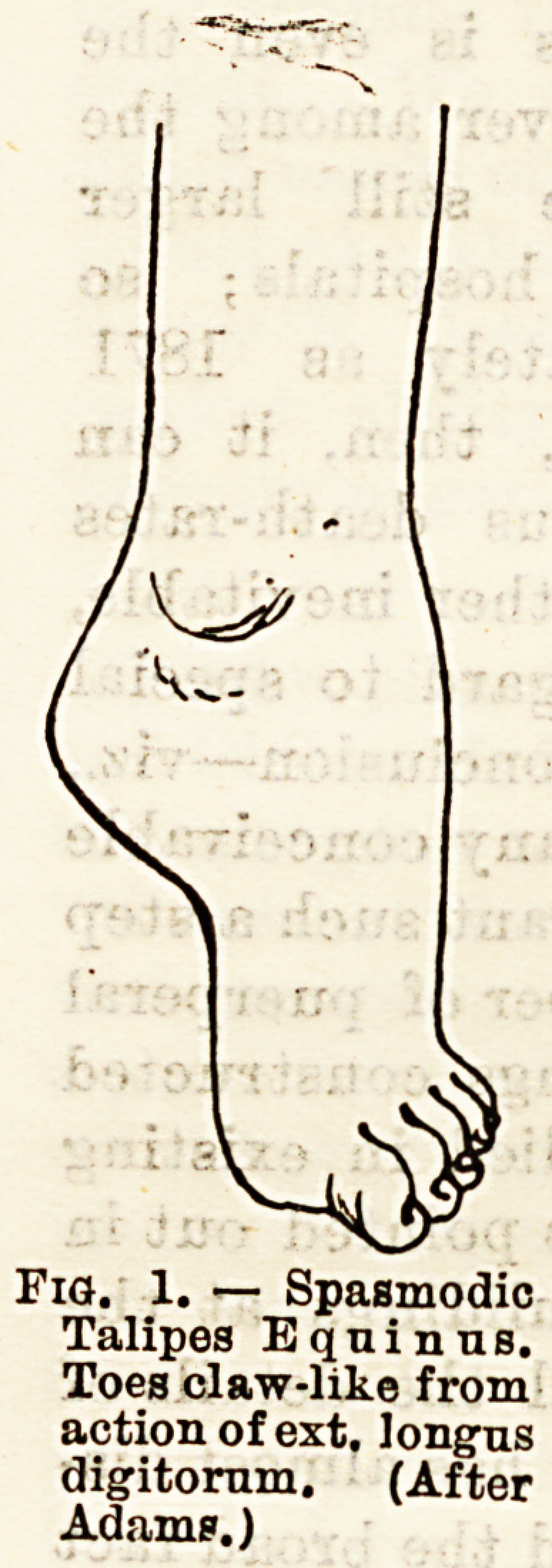


**Figs. 2 and 3 f2:**
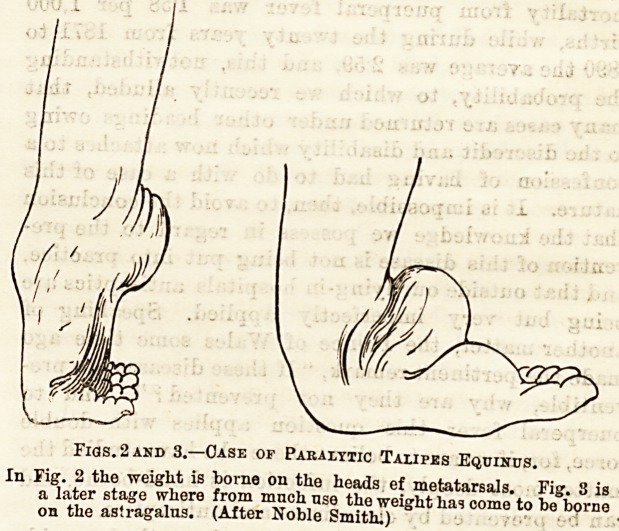


**Fig. 4 f3:**
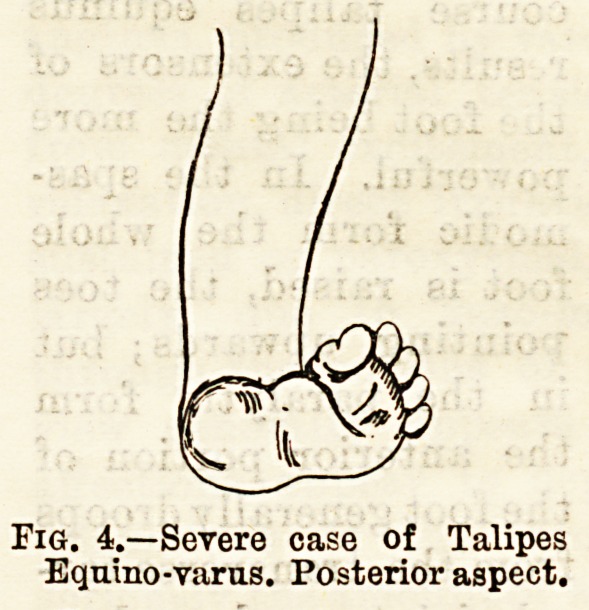


**Fig. 5 f4:**
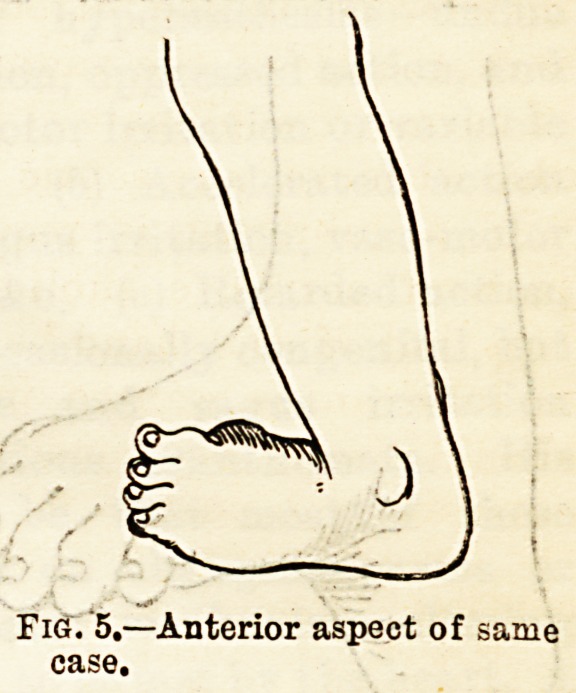


**Fig. 6 f5:**